# Invasive Meningococcal Disease—Changes in Epidemiologic Trends and Outcome over 24 Years in a Tertiary Care Hospital from Romania

**DOI:** 10.3390/pathogens14111083

**Published:** 2025-10-24

**Authors:** Constanța-Angelica Vișan, Miruna Norocea, Mădălina-Elena Neniu, Anuța Bilașco, Magdalena Vasile, Diana-Elena Vișan, Andreea Ioana Tudor, Anca Cristina Drăgănescu, Ana Maria Tudor

**Affiliations:** 1 Faculty of Dentistry, “Carol Davila” University of Medicine and Pharmacy, 050474 Bucharest, Romania; angelica.visan@umfcd.ro (C.-A.V.); anuta.bilasco@drd.umfcd.ro (A.B.); 2 “Prof. Dr. Matei Balş” National Institute of Infectious Diseases, 021105 Bucharest, Romania; noroceamiruna@gmail.com (M.N.); neniu.madalina@yahoo.com (M.-E.N.); magda.vasile@gmail.com (M.V.); tudor.andreea0711@gmail.com (A.I.T.); anca.draganescu@umfcd.ro (A.C.D.); 3 “Prof. Dr. N.C. Paulescu” National Institute for Diabetes, Nutrition and Metabolic Diseases, 020475 Bucharest, Romania; diana-elena.visan@rez.umfcd.ro; 4 Faculty of Medicine, “Carol Davila” University of Medicine and Pharmacy, 050474 Bucharest, Romania

**Keywords:** invasive meningococcal infection, serogroup, epidemiologic trends, Romania

## Abstract

**Introduction:** Despite the advances in its prevention, invasive meningococcal disease (IMD) remains a serious health problem worldwide due to its high morbidity and mortality, including in Romania, with 22% mortality rate. The objectives were to describe the trends of IMD cases admitted to a tertiary care hospital in Romania, over 24 years, and the case fatality rate (CFR) by age, year of admission, and *Neisseria meningitidis* serogroups. **Materials and Methods:** A retrospective study was conducted in IMD patients admitted to the National Institute for Infectious Diseases “Prof. Dr. Matei Bals”, Bucharest, Romania, between 2001 and 2024. **Results:** A total of 256 IMD patients were admitted, 171 under fourteen years, out of which 75 were infants. Most cases were reported before 2008. The case distribution showed 90 patients (35%) with meningitis, 84 (32%) with meningococcaemia, and 82 (32%) with an association of both. Serogroup B was the most frequently and steadily found (58 cases). The overall CFR was 12%. CFR was highest in infants younger than 6 months (19%) and meningococcaemia cases (29%). **Conclusions:** In our study, IDM had the highest frequency and CFR in infants and meningococcaemia cases. The trends showed a decline in cases after 2008. Serogroup B was the most prevalent and stable strain over 24 years.

## 1. Introduction

Invasive meningococcal disease (IMD), caused by *Neisseria meningitidis*, still remains a serious health problem worldwide due to its high morbidity and mortality, despite the recent advances in prevention [1].

*Neisseria meningitidis* is a Gram-negative coccus, almost always encapsulated, growing best on chocolate or blood agar and in a humidified 10% carbon dioxide environment. The colonies are bluish-gray and produce β-hemolysis after 48 to 72 h of incubation [2]. Meningococci are divided, for epidemiologic reasons, into serogroups and subtypes, based on the different polysaccharides in their capsules. There are twelve recognized serogroups: A, B, C, H, I, K, L, X, Y, Z, W-135 and 29E. Recently, serogroup D was reclassified as a variant of serogroup C [3].

IMD can cause meningitis, meningococcaemia, or an association of both, and it is characterized by a rapid progression, from variable and nonspecific symptoms to severe, life-threatening consequences within hours. IMD is associated with a mortality rate ranging between 5 and 10% in high-income countries [4].

In the absence of the timely initiation of the appropriate treatment, IMD mortality may exceed 50%, and even appropriate treatment is often insufficient to avoid severe sequelae among survivors. These sequelae include limb amputation, hearing loss, neurological deficits, skin scars, and emotional trauma for patients and their families [5].

The most effective preventive strategy to reduce morbidity and mortality due to IMD is vaccination. In 2005, the first quadrivalent meningococcal conjugate vaccine, MenACWY, was licensed [6]. The Advisory Committee on Immunization Practice (ACIP) recommended this vaccine for routine use in the United States, in adolescents aged 11–18 years. In 2014 and 2015, two vaccines for the serogroup B of *Neisseria meningitidis* were licensed and approved for use in young adults aged 10–25 years [7].

Immunization with the meningococcal conjugate vaccine for serogroup C (MenC) has resulted in a significant decrease in the incidence of meningococcal disease (MD) in many countries. A study carried out in Barcelona, Spain, showed an important decrease in MD incidence from 3.36 cases per 100,000 inhabitants in 1988, to 1.12 cases in 2015, while the serogroup B of meningococci remained the most prevalent in the above mentioned Spanish city, with an incidence of 1.22 per 100,000 inhabitants between 1997 and 2000, and 0.91 afterwards (2001–2007) [8]. Currently, both types of vaccines, for MenACWY and for MenB, are included in the immunization schedule for Spanish children starting from October 2015 [8,9].

In Europe, the increased number of IMD cases caused by MenC that occurred in the early 2000s was countered by the conjugate MenC vaccine, which was introduced in the national immunization program (NIP) in many countries. The rate of IMD cases in 29 reporting European countries dropped from 1.9 per 100,000 populations in 1999, to 1.1 in 2007, as a European Centre for Disease Control (ECDC) report showed [10].

Another European country, the Netherlands, introduced the MenC vaccine in 2002, for children aged 14 months, and a catch-up campaign for all the children up to the age of 18. As a result of this program, IMD caused by serogroup C was nearly eliminated [11]. After 2014, the number of IMD cases caused by serogroup W increased significantly and the MenC vaccine was replaced by the MenACWY quadrivalent conjugate vaccine, which is included in the NIP [11].

The meningococcal vaccination is included in the national immunization program in many countries around the world, and IMD is nowadays considered a vaccine-preventable disease.

In 2020, at the 4th summit of Global Meningococcal Initiative, the key discussion points were focused on three themes: the impact of the COVID-19 pandemic on epidemiology and vaccination programs of meningococcal disease, the growing trend of antibiotic resistance of *Neisseria meningitides*, and the broad coverage of meningococcal vaccines [12].

In 2023, the Federal Drug Administration (FDA) approved the use of a new pentavalent vaccine: MenABCWY for adolescents and young adults aging between 10 and 25 years. This pentavalent vaccine, included with a simplified schedule into the NIP, offers the possibility of broad vaccination coverage for IMD [13].

The incidence of IMD is still low and disease trends have decreased all over the world due to the efforts for vaccination, but, since 2021, a significant rise in the number of cases was recorded, surpassing the level recorded before the COVID-19 pandemic. In 2024, 503 cases of IDM were recorded in the US, probable and confirmed, representing the highest incidence registered since 2013. The main cause of this resurgence in cases was determined to be serogroup Y *Neisseria meningitides* [14].

In Romania, from 2010 until 2019, the incidence of IMD ranged between 0.26 and 0.34 per 100,000 individuals, for all ages and all serogroups [15]. The mortality rate caused by invasive meningococcal disease was over 22% in our country, as shown in the report from the National Center for Communicable Diseases Surveillance and Control issued in 2015 [16].

Since 2023, in Romania, only immunocompromised patients, as well as those over 65 years of age can receive free immunization. For other categories of the population meningococcal vaccination is optional, with a personal contribution [16].

The relevance of the paper is supported by the complexity of the epidemiological and clinical data, recorded over the 24-year period, in a country where the circulation of *Neisseria meningitidis* has not been significantly influenced by vaccination. The results of our study contribute more information regarding a serious, but preventable infectious disease, that can be useful to Romanian health authorities for future prophylactic programs.

Objectives:

The aim of the study was to describe the trends in IMD cases admitted to a tertiary care hospital in Romania by age, gender, different clinical forms, and meningococcal serogroup during a 24-year period.

The secondary objective of the study was to determine the fatality rate by age, gender, year of admission, and serogroup of the isolated strains of *Neisseria meningitidis* among the studied patients.

## 2. Materials and Methods

A retrospective observational study was conducted, including patients with the final diagnosis of confirmed or probable IMD admitted between 1 January 2001 and 31 December 2024 to the National Institute for Infectious Diseases “Prof. Dr. Matei Bals”, a tertiary care hospital in Bucharest, Romania.

The cases were selected from electronic medical records, using the diagnosis at discharge, including any of the following keywords: meningitis, meningococcaemia, sepsis, and *Neisseria meningitidis.* The analyzed data included age at admission, gender, date of admission, clinical status at discharge, and the identified serogroup.

The patients‘ ages were aggregated in nine age groups, as follows: the first two groups were for infants less than 6 months old, and 7 to 11 months old; and the other three groups were for children, including toddlers younger than 3 years old, preschool children (3–5 years old), and primary school children (6–14 years old). The rest of four adults’ groups included patients between 15 and 30 years old, 31 and 45 years old, 46 and 60 years old, and over 60 years old.

The definitions for the different types of meningococcal infections established by the Centers for Disease Control and Prevention were used. Confirmed IMD was defined using isolation in cultures or detection via the polymerase chain reaction (PCR) of *Neisseria meningitidis* in a sample of fluid from normally sterile sites (e.g., cerebrospinal fluid or blood).

Probable IMD was defined by clinical presentation compatible with IMD in the absence of microbiological or molecular/genetic confirmation (e.g., purpura fulminans).

*N. meningitidis* identification was performed using bacteriological methods, examination of Gram-stained smears, latex agglutination reaction, and genetic methods. The bacterial strains isolated in cultures were sent to the National Institute of Public Health and National Reference Laboratory, where the serogroups were confirmed with specific sera [17].

In Romania, the notification of IMD cases to the National Surveillance Center is mandatory and includes the reporting of probable and confirmed cases, as well as sending isolates for extensive typing [17].

The clinical conditions identified in studied patients were as follows: meningitis, meningococcaemia or meningococcal sepsis, and the association of both. Meningitis was defined as abnormal findings in cerebrospinal fluid, *Neisseria meningitidis* positive culture or polymerase chain reaction, or pleocytosis (more than 20 neutrophils/L) associated with suggestive clinical signs (e.g., fever, stiff neck, and rash).

Meningococcaemia was sustained by the detection of *Neisseria meningitidis* in blood by culture or PCR, associated with purpura, but with no clinical or microbiological criteria for meningitis present.

Association of meningitis with meningococcaemia was diagnosed based on microbiological findings in cerebrospinal fluid and/or blood associated with clinical suggestive symptoms for meningitis and meningococcaemia (e.g., fever, stiff neck, purpura, hypotension, and multiple organ dysfunctions).

The outcome included the following three categories: survivor, death, and lost to follow-up. In the last category, we included the patients transferred from our hospital to other facilities, whereupon no information about the final outcome, survival, or death was available.

The fatality rate was calculated for whole studied group, each age group, three clinical forms and identified serogroups.

*Statistical analysis*:

Descriptive indicators were presented as frequencies for categorical variables and mean, median, and interquartile intervals (IQR) for continuous variables. The *p*-value was computed using Fisher’s exact test, and the chi-square value was calculated for categorical variables using Mantel–Haenszel. A *p*-value of less than 0.05 was considered statistically significant. Univariate analysis for clinical manifestations of meningitis, meningococcaemia, or both was performed to determine distribution by age, gender, serotype and outcome. We used Epi Info 7 for Microsoft Windows Open Epi, Version 3, open-source calculator from the Centers for Disease Control and Prevention (CDC), website at https://www.cdc.gov/epiinfo, last accessed on 8 October 2025.

Figure plotting for numerical variables distribution was performed using Excel software, Microsoft Office Professional Plus 2019 software, and the diagram box-and-whiskers and violin plots, generated by Med Calc software (version 23.1.7, MedCalc Software Ltd., Ostend, Belgium) were used to evince the median values, dispersion of the groups, and the interquartile range. The evolution in time of the variables was represented using multiple line graphs with trends lines, generated by Med Calc.

## 3. Results

### 3.1. Study Population Characteristics

A total of 256 patients were admitted with the diagnosis of invasive meningococcal infection, and most of the cases, 171 (67%), were children younger than fourteen years. The infants, less than one year old, were the most affected age group—75 out of 256 cases (29%).

In children, the mean age was 2.6 years (SD ± 3.5), while among adults the mean age was 38 years (SD ± 19.1), and 14 years (SD ± 20.3) was the mean age in the whole group. The youngest patient was 22 days old at admission and the oldest patient was 88 years old.

Median age was 1 year old in children, ranging from 1 month to 14 years (IQR: 0.58–3), and 34 years in adults, from 16 to 84 years (IQR: 21–54) (Table 1).

### 3.2. Age Groups Distribution Analysis

**Figure 1:**
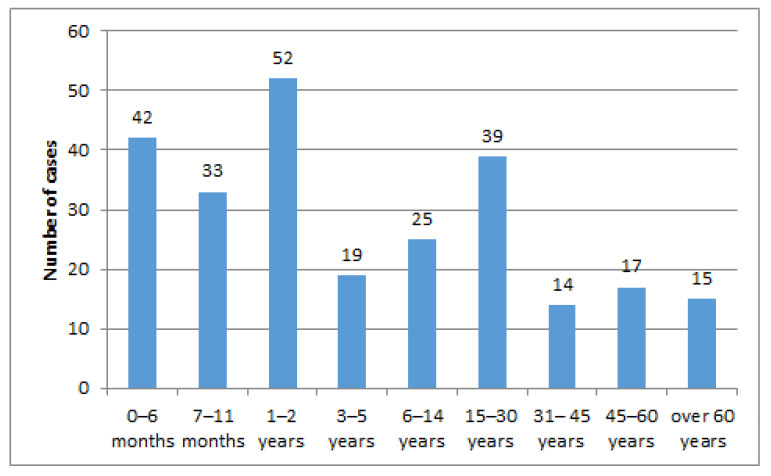
Case distribution by age groups.

The distribution of the age groups in studied patients, plotted in Figure 1, showed that the highest number of cases was observed in infants, 75 (30%), followed by toddlers less than tthree years, 52 (20%), as well as adolescents and young adults aged fifteen to thirty years old, 39 (15%), respectively. The lowest number of cases was noticed in adults thirty-one to forty-five years old, 14 cases (5%) and over sixty years old, 15 cases (6%).

Gender distribution, 130 males (51%) and 126 females (49%), was homogeneous distributed in all the studied patients, and was consistent in children (sex ratio M/F = 85/86) and adults (sex ratio M/F = 45/40) (Mantel–Haenszel chi-square = 0.2366, *p*-value = 0.7231) (Table 1).

Microbiological confirmation of *N. meningitidis* infection was obtained in 219 cases (86%), with no statistically significant differences in confirmation rates among children and adults, 145 (85%) and 74 (87%), respectively (Mantel–Haenszel chi-square 0.2344, *p*-value = 0.7781).

Clinical manifestation of meningitis, meningococcaemia, and association of both was found in 90 patients (35%), 84 (33%) and 82 (32%) cases, respectively, showing an even distribution within the whole group. Analysis of the IMD studied clinical forms by age revealed that the number of children diagnosed with meningitis was significantly lower compared to adults with the same diagnosis (Mantel–Haenszel chi-square 9.511, *p*-value = 0.00339).

Meningococcaemia was more frequent in children versus adults, and the difference was statistically significant (Mantel–Haenszel chi-square 6.29, *p*-value = 0.01612). At the same time, the association of meningitis and meningococcaemia affected children and adults at similar rates (Mantel–Haenszel chi-square = 0.3995, *p*-value = 0.6267).

Serogroups were identified in 90 cases (35%). Most of the cases in the studied group had infection with *Neisseria meningitidis* serogroup B, 58 out of 90 strains (64%), followed by serogroup C, with 21 strains (23%), serogroup A, with 7 strains (8%), serogroup Y in two cases (2%), and one case each of W and D strains (1%).

Upon analyzing the different serogroups distribution in the studied population by age, it was observed that serogroups B (Mantel–Haenszel chi-square 0.421, *p*-value = 0.6730), C (Mantel–Haenszel chi-square = 0.007179, *p*-value = 0.9999), and A (*p*-value = 0.8800, chi-square not recommended due to low number of cases) were not significantly more frequent among children versus adults. A similar result was noted for distribution of undetermined serogroup cases, with 116 children (68%) and 50 adults (59%), respectively (Mantel–Haenszel chi-square = 2.015, *p*-value = 0.2002). For the serogroups D, Y, and W, the statistical analysis was not performed due to the low number of cases found.

The outcome distribution had no statistically significant differences in children compared to adults (Mantel–Haenszel chi-square 0.2756, *p*-value = 0.7596) in our study. The case fatality rate in children was 22 out of 171 patients (13%), while in adults, the case fatality rate was 9 out of 85 cases, (11%). The overall case fatality rate was 31 out of 256 (12%).

### 3.3. Clinical Manifestations

In our study, the three clinical categories, meningitis, meningococcaemia and association of both, had a similar distribution by age group, as is shown in Table 2.

Among patients younger than 14 years old, there is no statistically significant difference in clinical manifestation, meningitis, meningococcaemia or both, by age group (Mantel–Haenszel chi-square = 3.9935, *p*-value = 0.857706). Similar results were observed in clinical manifestation distribution in patients older than 14 years (Mantel–Haenszel chi-square = 4.9402, *p*-value = 0.551507).

Gender distribution between three categories of clinical manifestation was not statistically significant (Mantel–Haenszel chi-square = 1.8598, *p*-value = 0.394603).

The number of deaths among meningitis cases was low, at 2 out of 90 patients (2%), among meningococcaemia cases it was registered 24 deaths out of 84 (29%) patients, and in the case of the association of both clinical manifestations, 5 deaths out of 82 patients (6%).

The case fatality rate for the whole group of patients was 12%. A statistically significant difference (Mantel–Haenszel chi-square = 23.59, *p*-value = 0.0000874, RR 0.1288, 0.03378–0.4912) was found comparing the fatality rate among patients with meningococcaemia, 29%, and those with meningitis, 2%, showing that in patients diagnosed with meningococcaemia alone, the risk of death was higher compared to those diagnosed with meningitis alone. Furthermore, the fatality rate among meningococcaemia cases was statistically significant higher versus patients with meningitis plus meningococcaemia, namely 29% and 6%.(Mantel–Haenszel chi-square = 14.45, *p*-value = 0.0001161, RR 1.89, 1.469–2.432).

When evaluating the case fatality rate among children with meningococcaemia, which was 31% (20 out of 65 cases), and children with meningitis (no death recorded), and the association between meningococcaemia and meningitis, which was 3.5% (2 out of 57 cases), we found a statistically significant difference (Mantel–Haenszel chi-square = 29.81, *p*-value < 0.0000001), showing that the risk of death was higher in children without meningeal involvement.

The same analysis in adults showed that the fatality rate among the meningococcaemia cases, at 21% (4 out of 19 cases), was not statistically significantly higher compared to the fatality rate in meningitis alone, at 5% (2 out of 41 cases) (Mantel–Haenszel chi-square = 3.711, *p*-value = 0.1471), and the fatality rate among the cases of association of meningitis and meningococcaemia, at 12% (3 out of 25 cases) (Mantel–Haenszel chi-square = 0.3085, *p*-value = 0.8815). In the studied adult population, the risk of death was not influenced by the presence of meningeal involvement.

The difference in meningococcaemia case fatality rate between children and adults was not statistically significant (Mantel–Haenszel chi-square = 0.6721, *p*-value = 0.6049), showing that the risk of death in severe invasive meningococcal disease was similar in children and adults.

Serogroups distribution among the studied clinical categories revealed no statistically significant difference (Mantel–Haenszel chi-square = 11.5203, *p*-value = 0.073567).

Among the studied meningococcaemia cases, the number of indeterminate serogroup strains was statistically significantly greater than the number of identified ones (Mantel–Haenszel chi-square = 7.109, *p*-value = 0.02860). In other clinical study groups, the low number of cases with identified serogroups did not meet the criteria for statistical analysis.

### 3.4. Outcome

Univariate analysis for outcome was performed considering the following: two age groups, children and adults; gender; three clinical manifestations, meningitis, meningococcaemia and association of both; and three serogroups, B, non-B and indeterminate. The results are presented in Table 3. Lost-to-follow-up patients, representing 5% of the whole studied group (12/256), were excluded from this analysis. The distribution of the lost-to-follow-up cases was as follows: six meningitis cases, five meningococcaemia cases, and one meningitis associated with meningococcaemia, with nine cases in children and three in adults, serogroup B and A with one case each, and ten unidentified strains.

Analysis of outcome distribution found no statistically significant differences between children and adults. The survival rate in children, at 140 out of 162 cases (86%), was similar with the rate in adults, at 73 out of 82 cases (89%) (Mantel–Haenszel chi-square = 0.3317, *p*-value = 0.5398) (Table 3).

The number of survivors among males was 113 out of 128 cases (88%) and in females, 100 out of 116 cases (86%). The distribution of the survival rate was similar among genders (Mantel–Haenszel chi-square = 0.2351, *p*-value = 0.6326).

The survival rate was not significantly different in males versus females in the three studied clinical categories, namely meningitis, meningococcaemia, and association of both (Mantel–Haenszel chi-square = 0.33481, *p*-value = 0.845879).

An analysis of the three categories of meningococcal serogroups, B, non-B and indeterminate, showed that the surviving rate was lower in the indeterminate serogroup cases (Mantel–Haenszel chi-square = 10.45, *p*-value = 0.005389), versus B and non-B serogroups. This result suggests that fatal cases were associated with difficulties in obtaining the laboratory confirmation of infection or the samples needed for extensive microbiologic determination, mainly due to the particular aspect of the disease, characterized by fulminant evolution, leading the medical team to focus on rapid treatment initiation, including antibiotics, rather than collecting biologic samples.

Univariate analysis demonstrated that the survival rate among meningococcaemia cases was significantly lower than that observed in the other two groups, meningitis and association of meningitis and meningococcaemia (Mantel–Haenszel chi-square = 33.44, *p*-value is <0.00001).

Upon further stratification of meningococcaemia alone cases by age group, statistically significant differences in survival rate were observed, with the subgroup of children aged one to two years displaying the lowest survival rate—52% (*p*-value = 0.0003737, RR = 8.049, OR = 27.27, Mantel–Haenszel chi-square not recommended due to low number of cases)—followed by infants—65% (21 out of 31 cases) (Mantel–Haenszel chi-square 6.221, *p*-value = 0.01263, RR 2.115, OR 4.29).

Univariate analysis among children under two years, including infants, revealed that the diagnosis of meningococcaemia alone was significantly associated with a lower survival rate compared to meningitis and association of both clinical manifestations diagnosis, suggesting that in this research, the presence of meningeal involvement was associated with a higher survival rate in children younger than two years old. The results of the univariate analysis in other age groups found no statistically significant differences in survival rate between meningococcaemia alone and aggregated meningeal involvement group, as presented in Table 4.

### 3.5. Invasive Meningococcal Disease Trends During the Studied Period

**Figure 2:**
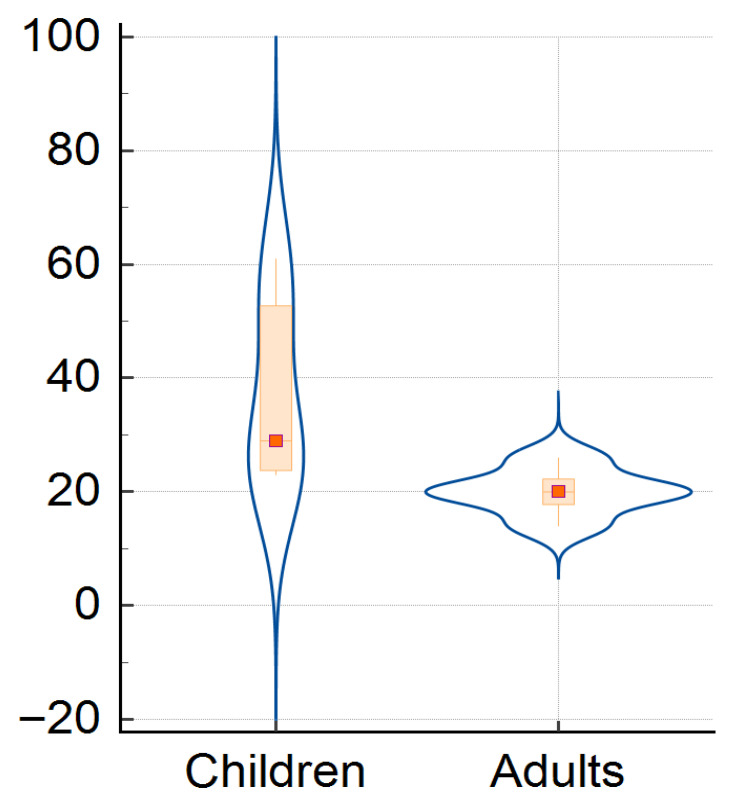
Distribution of children and adults’ cases for whole study period.

The children and adults‘ cases distribution over the study period is plotted in Figure 2, showing an asymmetric density of cases, meaning that children were the most affected group compared to adults, especially in the first studied decade, but this was not statistically significant (Mantel–Haenszel chi-square = 6.6, *p*-value = 0.252).

The mean number of cases per year during the studied period was 10.7 (SD+/− 7.3) and the median number of cases was 9.5 (IQR = 10.75).

In the first decade of the study period, the number of cases was higher than average, at 10.7 cases per year. The highest number of cases were recorded in 2003, namely 31 cases, representing 12% of all studied patients. In the next five years the number of IMD cases was relatively stable. After 2009, the total number of cases dropped sharply. This reduction in IMD cases admitted to our hospital could have different causes, like the addressability of the population and changes in admission criteria, among other factors, which are aspects not assessed by our study; therefore, a pertinent conclusion couldn’t be reached.

The very low number of cases observed in 2020 and 2021 coincided with the COVID-19 pandemic, when the admission to our hospital of other cases besides SARS-CoV-2 infection was very limited. An increasing number of IMD cases was noted starting in 2021, with an upward trend which continued till the end of study period.

**Figure 3:**
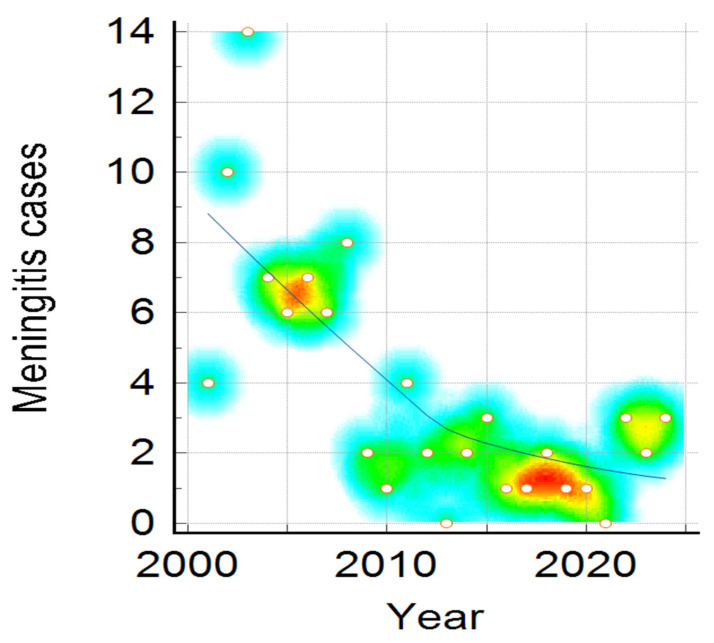
Meningitis Cases distribution during the studied period. Color intensity was associated with number of cases, the transition from warm to cool colors represents the variation from higher to fewer cases ( red, yellow, green and the lowest value—blue).

**Figure 4:**
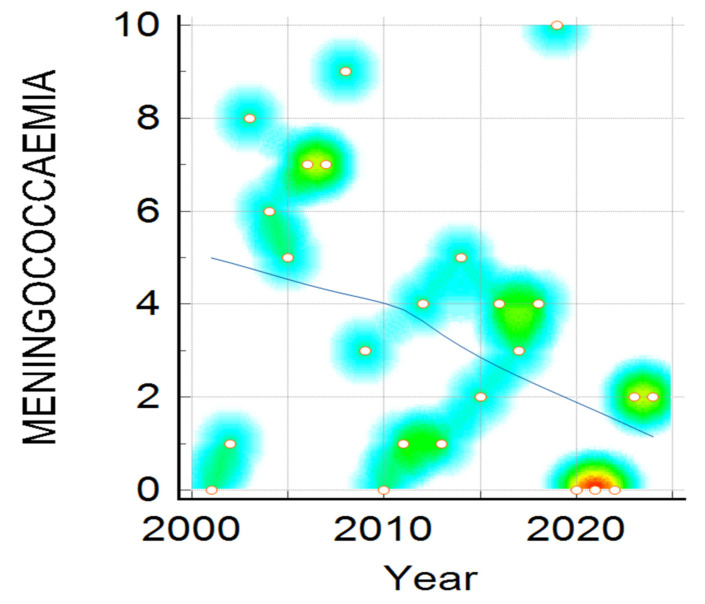
Meningococcaemia cases distribution during the studied period. Color intensity was associated with number of cases, the transition from warm to cool colors represents the variation from higher to fewer cases ( red, yellow, green and the lowest value—blue).

**Figure 5:**
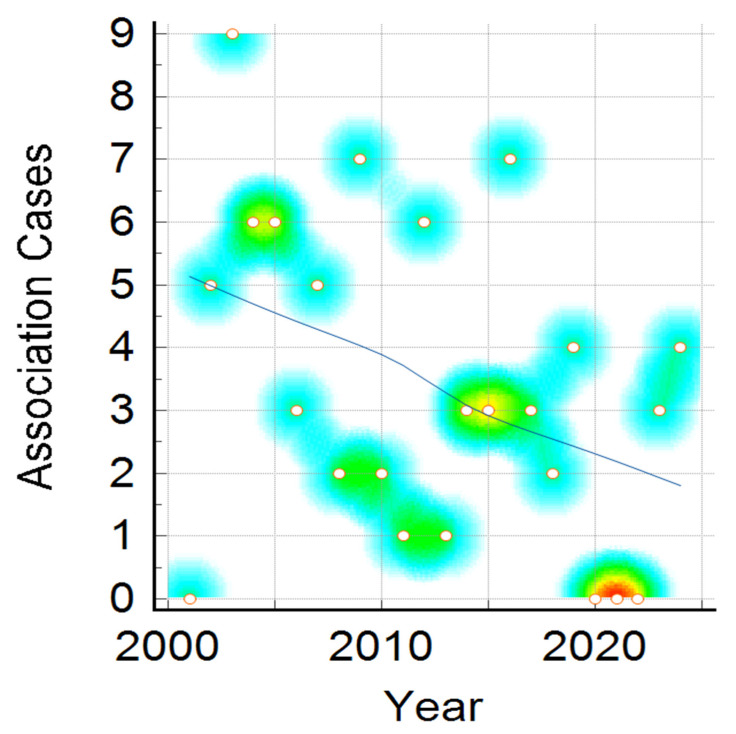
Association of meningitis and meningococcaemia cases distribution during the studied period. Color intensity was associated with number of cases, the transition from warm to cool colors represents the variation from higher to fewer cases ( red, yellow, green and the lowest value—blue).

Clinical presentation, namely meningitis, meningococcaemia, or association of both, distribution by year, shown in Figure 3, reveals a downward trend for all clinical forms, and is more visible in meningitis cases compared to meningococcaemia and in the association of meningococcaemia and meningitis cases. The decrease in number of IMD cases admitted to our hospital during the studied period was statistically significant in all three clinical categories (Mantel–Haenszel chi-square = 14.513, *p*-value = 0.005825), showing a noticeable change over 24 years (Figure 3, Figure 4 and Figure 5).

In Figure 3, it can be observed that the number of meningitis cases is higher around 2005 and 2008, plotted in red dots, followed by a downward trend till the end of the studied period, with an increase in number of cases in 2015.

Analyzing the trends in meningococcaemia cases, plotted in Figure 4, it can be noted there is a similar dispersion in the number of cases, which is higher in 2003, 2008, and again in 2019, following a similar declining trend.

Association of meningitis and meningococcaemia showed a similar decreasing number of cases in the 24-year period, with more cases in 2003–2005, 2012, 2019 and 2024, but in general with a descending trend, similarly to the previously presented trends of meningitis alone and meningococcaemia alone (Figure 5).

**Figure 6:**
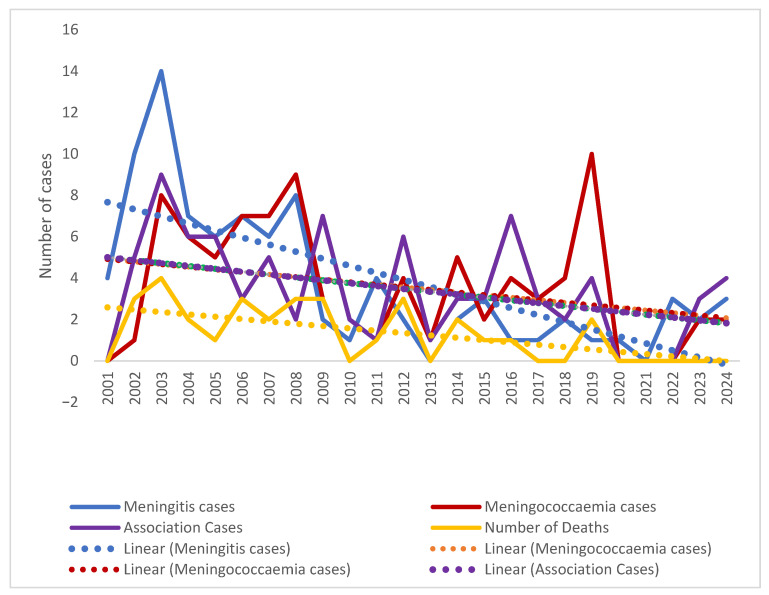
Trends in IMD clinical forms over the studied period (2001–2024).

At the same time, the distribution of the recorded fatal cases over the studied period had a similar drop, with no death recorded among IDM patients in the last four studied years. IMD case fatality rate changes during the studied period were not statistically significant (Mantel–Haenszel chi-square = 4.5622, *p*-value = 0.10217) (Figure 6).

**Figure 7:**
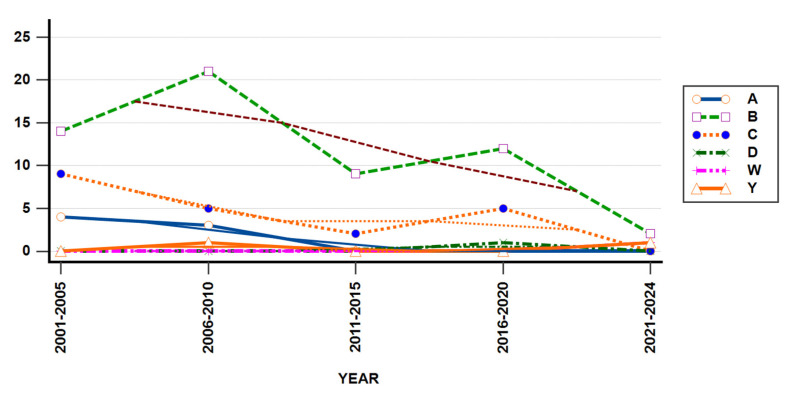
Serogroups trends in studied population from 2001 to 2024.

Meningococcal serogroup B was the most frequently identified strain, comprising 58 out of 90 strains (64%) during the whole studied period, compared to other identified meningococcal serogroups (Figure 7). MenC strains accounted for 23% (21/90 cases) of all identified strains and exhibited continuous presence in the first decade, except for three years period, from 2010 to 2013. They reemerged prior to the COVID-19 pandemic, but were not detected in the most recent five years.

*N. meningitidis* Serogroup A was detected throughout the first eight years, accounting for 7% of all identified meningococcal strains, and not found to circulate after 2008. MenY strains were a rare finding during the studied period, being detected in just two cases, in 2008 and 2024, respectively. In 2017, the serogroup D strain was identified in one studied patient. These results showed the constant presence of two main strains, B and C, the resurgence of MenY strain in 2024, the disappearance of MenA strain after 2008, and the emergence of a new strain, MenW, in 2024. Yearly IMD cases distribution showed similar patterns among different studied serogroups (Mantel–Haenszel chi-square = 5.231, *p*-value = 0.5231).

The changing trends of different meningococcal serogroups within the 24-year study period could have many causes, such as the following: the real replacement of a strain by another one, better laboratory testing, implementing new diagnostic guidelines, and the impact of vaccines’ introduction in our country at distinct time points. These were aspects that were not assessed in our study, and thereby we cannot ascertain any conclusions or explain the observed data.

## 4. Discussion

The particularity of the data obtained in this analysis results from the long period analyzed, of 24 years, and from the comparing of data between children and adults, which had not been performed before in our country, to the best of our knowledge. The long period analyzed provides superior data accuracy and information about different epidemiological aspects in relation to all age groups, and offers real-world insights about IMD treatment in Romanian hospitals.

It is important to mention the fact that meningococcal vaccines have been registered in Romania since 2017, but they are not yet included in the NIP in our country [18]. In this context, the data about meningococcal vaccination coverage were lacking from National Public Health Center reports, including from the last one in 2024 [19].

From this latest report, it was observed that Romania had a very low vaccination coverage, around 50%, for vaccines included in NIP in the last 10 years, suggesting an even worse situation for other preventable diseases, including meningococcal infection.

The Romanian National Surveillance System for Communicable Diseases mandates reporting probable and confirmed cases of IMD and sending isolates for extensive typing [16].

The largest group of patients with invasive meningococcal infection in our research was the group of infants (75/256), followed by children aged one to two years (52/256). This result can be explained by the low immunization coverage in the general population, especially in toddlers, starting from the first year of life. Moreover, these results are consistent with data from the literature all over the world [20]. The rate of IMD is the highest in children under 1 year of age, followed by the rate in children aged between 1 and 4 years old, and adolescents between 15 and 24 years old. In Europe, according to the report from 2022, the annual incidence of laboratory-confirmed cases was 0.3/100,000 for all, 4.3/100,000 for infants under 1 year of age, 0.8/100,000 for children between 1 and 4 years, 0.2/100,000 for children between 5 and 14 years, and 0.6/100,000 for adolescents and young adults (15 to 24 years). Although incidence rates are much lower in the United States (0.09/100,000 in 2022), the age distribution is similar [21].

At the same time, the high number of young patients in the present study had a great influence on mean age, of 14 years, for the whole studied group, and it was consistent with the observed median age of 1 year in children and 34 years in adults.

A similar study, conducted by Maturana and colleagues in a Spanish tertiary level hospital, found that the median age was 2 years in pediatric patients (IQR, 0.7–7.5) and 41.2 years in adult patients (IQR, 26.4–69.3) [8]. These differences in median age between the Spanish study and ours can be explained by a mismatched time frame and the variability in admission criteria between countries.

It was noted that there was a similar rate of laboratory-confirmed cases in this research to that observed in the previously mentioned Spanish study, at 85.54% (219 out of 256 cases) versus 85.7% (72 out of 84 cases), suggesting the fact that, despite the longer duration of our study, including periods before digital medical records, we managed to retrieve the information needed, and this gave us confidence in our data [9].

In this research, it was found that there was a significantly lower rate of children with meningitis and higher rate of meningococcaemia versus adult patients, results that were in contrast with the data published by Middeldorp and colleagues in 2023, from an epidemiological study carried out in the Netherlands from 2011 to 2020, showing that children under 5 years had a higher rate of meningitis, at 46%, compared to adults, while adults had higher rate of sepsis, at 38%, compared to children [11]. The divergent results can be explained by the non-identical time frame and patient selection. In our study, data was collected only from one hospital, not at a national level, as it was the case in the Netherlands’ epidemiological study.

Data from the present study showed an overall case fatality rate of 12%, which was similar to that reported in the literature. Reports from a Serbian IMD published article observed a case fatality rate, over a 28-year span, ranging from 7.5% to 21.4%, similar to the case fatality rate recorded in the study presented in this paper [22]. Similar data was reported by a study carried out in Canada, where the mortality rate was 12% in adults and 4% in children [23].

The case fatality rate in this research was significantly higher in patients diagnosed with meningococcaemia alone versus those diagnosed with meningitis alone. The data showed a similar rate of fatal outcomes in children with meningococcaemia compared to adults, at 31% versus 21%, respectively. In an epidemiological paper from Israel, during a twenty-year period, Ben-Shimon and colleagues observed a higher mortality in children with meningococcaemia versus meningitis, but at a lower level, at 14.9 vs. 7.9%, compared to our data [24].

Moreover, meningococcaemia’s fatality rate in children versus adults was not significantly different in this analysis. The data reported by researchers in other European countries varied; for example, Contou reported that in France the highest CFR was 37% in adults older than 65 years with purpura fulminans, while in the same country, Floret reported a CFR of 79% in pediatric population with purpura fulminans [25,26]. These discordant results can be explained by the selection criteria of the patients, with only IMD cases admitted in one tertiary care hospital, as opposed to collecting the data from the entire country, or utilizing definitions for the clinical forms, like analyzing just the most severe form of the IDM, purpura fulminans.

*Neisseria meningitidis* serogroup B was the most frequent strain observed among our cases, with a total of 58 out of 90 strains (64%), followed by MenC strains, both of them being present almost every year during the studied period. MenA strains represented 8% of all identified serogroups in this study; however, the last case of IMD caused by serogroup A was identified in 2008.

There was a significant number of cases caused by MenC and MenA, which is probably due to the lack of vaccination in the first year of age. In many countries there are not many cases of IMD with MenA between 2010 and 2020, as Tzanakaki and coleagues mentioned in a study recently published [27]. The presence of IMD cases with serogroup A in our country was also noted by the above-mentioned authors in a paper focusing on the IMD situation in south-eastern Europe [27]. They observed that Romania was one of the few countries that reported cases due to serogroup A, although the numbers of cases were small. The number of cases of IMD due to serogroup B and C have decreased in our country, as well, and cases due to serogroups W or Y have become uncommon [27].

It is noticed that MenY strains were a rare finding, with just two cases recorded in 2008 and 2024. At the same time, the MenW strain, not seen before, emerged in the last studied year. MenY and W are the two new serogroups circulating in Europe due to epidemiological changes associated with vaccine approval in various countries [28,29].

An analysis of IMD epidemiology over a 10-year period in Europe revealed distinct trends, despite the well-known unpredictability of this disease. Between 2008 and 2017, the incidence of IMD decreased overall by 34.4% and there were significant shifts across serogroups in different countries and for different age groups [30]. The greatest concerns were related to the increase in the incidence of serogroups W and Y at all ages, as well as the increase in the number of cases, regardless of serogroup, in the elderly population [30].

Recently, the incidence of IMD caused by serogroup Y and W increased all over the world, reaching up to 50% of IMD in some European countries. In Spain, the number of cases of IMD caused by Men W increased 4-fold between 2015 and 2016, compared to previous years [9]. In France, the National Reference Center for Meningitis at the Pasteur Institute have noticed the emergence of MenW, which was linked to the Haji pilgrimage of 2000, and the expansion of isolates belonging to the South American–UK strain, since 2013 [31].

Serogroup W remained the second most common cause of IMD and was reported in 12% of serogroup documented cases, followed by serogroup C (12%) [32]. We did not notice this situation in our study due to the low number of cases of serotyped strains.

Routine meningococcal immunization performed in many countries in children and adolescents has markedly reduced the burden of IMD. As a consequence, the proportions of IMD cases in adults over 60 years of age, who remain unvaccinated, have increased in these countries, comprising up to almost 25% of all cases [33]. Among these cases, mortality is highest and survivors often experience long-term sequelae. IMD due to serogroups W and Y are more common and clinical manifestations may be atypical, such as pneumonia and gastrointestinal symptoms, which can delay the diagnosis and treatment. For this reason, the need for policy makers to provide meningococcal vaccines for these age groups is significant [33].

In Romania, although the meningococcal vaccination is not included in the NIP, free vaccination for people over 65 years of age is available. The epidemiological trends of IMD has changed over the years worldwide, including Romania, creating a continuous need of data for the cost-effective implementation of prevention. There are several vaccines available for IMD prevention worldwide, specific to some serogroups, so data from studies like ours can provide useful information to national authorities regarding the serogroups actually circulating in the Bucharest area, and the burden of the disease in this region [18,34].

Analyzing the trends in IMD cases over 24 years, it was found that the period with the highest number of hospitalized cases was recorded between 2003 and 2009, with a peak in 2003, followed by a significant decrease in recent years. This situation can be explained by the trends of circulating strains in the neighboring countries and in Europe in general, as well as by the efficacy of the prevention measures, diagnosis, and treatment guidelines implemented in our country.

An article that was recently published in *Vaccine* journal presented a report of IMD cases registered in Vojvodina, Serbia, over a 28-year period, between 1997 and 2025. In this nearby region of Serbia, the authors reported the peak of the annual incidence to be in 1997 (1.24/100,000). They have noticed smaller surges in 2003 and 2005, and the last peak of 175 cases, in 2006. A slight resurgence occurred in 2023–2024, with 13 cases reported. Our results are consistent with Serbian data, showing an important reduction in the number of cases. Moreover, we found an important decrease in case fatality rate in this research [22].

### Study Limitations

Our study design introduced some limitations. Firstly, data was collected retrospectively, and this aspect posed a higher risk of not being able to find some information. Secondly, the data were from a single tertiary hospital in our country. Although it is one of the most important hospitals of its kind, collecting data from more hospitals could provide a clearer picture of the IMD situation in our country. It also could be the case that a greater proportion of severe cases were directed to us. Most cases were transferred to our clinic from other hospitals in the country, already having received antibiotics and sometimes without complete clinical and laboratory data, which explains the number of probable cases. In addition, the length of our study period, more than twenty years, brought the same challenges due to the improvement in the laboratory capacity for identifying serogroups, the digitalization of medical records, and the changes in clinical guidelines for diagnosis and treatment. Finally, the high proportion of indeterminate serogroups had an important influence in the data analysis. The results of our long period study may not reflect the exact reality of the epidemiological situation of IMD in our country, but can reflect the trends in severe cases of IMD, and the fatality rate for pediatric and adult populations, offering the opportunity to update and adapt the local guidelines for meningococcal infection diagnosis, treatment, and prevention in different age groups.

## 5. Conclusions

Infants and meningococcaemia cases had the highest frequency and fatality rate during the 24-year study period. However, the number of fatal cases declined in last ten years of the study. *N. meningitidis* serogroup B was the most prevalent strain and maintained a relatively flat trend over the 24-year study, compared to other identified serogroups. In the same time frame, MenB infections case fatality rates remained stable, suggesting a constant virulence of the serogroup B strains.

Surveillance of meningococcal disease is essential in order to monitor changes in disease incidence, to evaluate the burden of IMD, and to make recommendations for health authorities concerning vaccine policy for IMD prevention in our country.

The introduction of meningococcal vaccination in the national immunization program from the first year of life can reduce the burden of this disease in our country.

## Figures and Tables

**Table 1:** Patients’ characteristics.

Characteristic	Children (N= 171)	Adults (N = 85)	Total (N = 256)	*p*-Value
Age (years)	2.6	38.3	14.4	
Mean (SD)	(±3.5)	(±19.1)	(±20.3)	
Median	1	34	3	
(range)	(1 month–14 years)	(16–84 years)	(1 month–84 years)	
IQR	0.58–3	21–54	0.81–21	
Gender M%	85 (50%)	45 (53%)	130 (51%)	0.7231
F%	86 (50%)	40 (47%)	126 (49%)	
Laboratory-confirmed IMD (Yes/No)	145 (85%)	74 (87%)	219 (86%)	0.7781
	26 (15%)	11 (13%)	37 (14%)	
Type of manifestation				
Meningitis	49 (29%)	41 (48%)	90 (35%)	0.00339
Meningococcaemia	65 (38%)	19 (22%)	84 (33%)	0.01612
Meningitis and + Meningococcaemia	57 (33%)	25 (30%)	82 (32%)	0.62667
Serogroup				
B	34 (20%)	24 (28%)	58 (23%)	0.6730
C	13 (8%)	8 (9%)	21 (8%)	0.9999
A	5 (2.5%)	2 (2%)	7 (3%)	0.8800
Y	2 (1%)	0	2 (1%)	Not done
W	1 (0.5%)	0	1 (0.5%)	Not done
D	0	1 (1%)	1 (0.5%)	Not done
Not determined	116 (68%)	50 (59%)	166 (65%)	0.2002
Outcome				0.7596
Death	22 (13%)	9 (11%)	31 (12%)	
Survivors	140 (82%)	73 (86%)	213 (83%)	
Lost to follow-up	9 (5%)	3 (4%)	12 (5%)	

**Table 2:** Clinical categories distribution by age, serogroup, and outcome.

Parameter	Meningitis Cases (N = 90)	Meningococcaemia Cases (N = 84)	Meningitis + Meningococcaemia (N = 82)	*p*-Value
Age group				
Children				0.857706
0–6 months	12 (13%)	17 (20%)	13 (16%)	
7–11 months	8 (9%)	14 (17%)	11 (12%)	
1–2 years	16 (18%)	21 (25%)	15 (18)	
3–5 years	6 (7%)	7 (8%)	6 (7%)	
6–14 years	7 (8%)	6 (7%)	12 (15%)	
Adults				0.551507
15–30 years	17 (19%)	11 (13%)	11 (13%)	
31–45 years	10 (11%)	1 (2%)	3 (4%)	
46–60 years	8 (9%)	4 (5%)	5 (6%)	
over 60 years	6 (7%)	3 (4%)	6 (7%)	
Gender M (%)	50 (56%)	38 (45%)	42 (51%)	0.394603
Serogroup				0.073567
B	20 (22%)	11 (13%)	27 (33%)	
C	7 (8%)	7 (9%)	7 (9%)	
A	6 (7%)	0	1 (1%)	
Y	0	1 (1%)	1 (1%)	
W	0	1 (1%)	0	
D	0	1 (1%)	0	
Not determined	57 (63%)	63 (75%)	46 (56%)	
Outcome Fatal cases Survivors Lost to follow-up	2 (2%) 83 (92%) 5 (6%)	24 (29%) 54 (64%) 6 (7%)	5 (6%) 76 (93%) 1 (1%)	<0.00001

**Table 3:** Outcome distribution by age, gender, clinical manifestation, and serogroup.

Parameter	Number of Fatal Cases (N = 31)	Number of Survivors (N = 213)	*p*-Value
Age			0.5798
Children	22/162 (14%)	140/162 (86%)	
Adults	9/82 (11%)	73/82 (89%)	
Gender			0.6326
Male	15/128 (12%)	113/128 (88%)	
Female	16/116 (14%)	100/116 (86%)	
Clinical manifestation			<0.00001
Meningitis	2/84 (2%)	82/84 (98%)	
Meningococcaemia	24/79 (30%)	55/79 (70%)	
Both	5/81 (6%)	76/81 (94%)	
Serogroup			0.005389
B	2/57 (3.5%)	55/57 (96.5%)	
Non-B	1/30 (3%)	29/30 (97%)	
Indeterminate	28/157 (18%)	129/157 (82%)	

**Table 4:** Survival rate distribution by age group and diagnosis (meningococcaemia versus meningeal involvement).

Age Group	Meningeal Involvement cases (N = 172)	Number of Surviving Cases with Meningeal Involvement	Meningococcaemia Alone Cases (N = 84)	Number of surviving Cases with Meningococcaemia Alone	*p*-Value
0–11 months	44 (26%)	39/44 (89%)	31 (37%)	20/31 (65%)	0.01263
1–2 years	31 (18%)	31/31 (100%)	21 (25%)	11/21 (52%)	0.0003737
3–5 years	12 (7%)	12/12 (100%)	7 (8%)	5/7 (71%)	0.5924
6–14 years	19 (11%)	19/19 (100%)	6 (7%)	3/6 (50%)	0.06245
15–30 years	28 (16%)	26/28 (93%)	11 (13%)	9/11 (82%)	0.6259
31–45 years	13 (8%)	11/13 (85%)	1 (1%)	1/1 (100%)	0.7429
46–60 years	13 (8%)	12/13 (92%)	4 (5%)	3/4 (75%)	0.8529
over 60 years	12 (3%)	9/12 (75%)	3 (4%)	2/3 (67%)	0.4108

## Data Availability

The datasets supporting our research published here are available on request from the corresponding authors.
